# Effect of manipulative reduction combined with air enema on intestinal mucosal immune function in children with intussusception

**DOI:** 10.12669/pjms.36.7.3105

**Published:** 2020

**Authors:** Yang Li, Han-liang Jiao, Yu-kun Bai, Ping Wang

**Affiliations:** 1 Yang Li, Department of Minimally Invasive Surgery, Hebei Children’s Hospital, Shijiazhuang, 050031, Hebei, P.R. China; 2 Han-liang Jiao, Department of Pediatrics, Hebei Children’s Hospital, Shijiazhuang, 050031, Hebei, P.R. China; 3 Yu-kun Bai, Department of Pediatrics, Hebei Children’s Hospital, Shijiazhuang, 050031, Hebei, P.R. China; 4 Ping Wang, Department of Pediatrics, Hebei Children’s Hospital, Shijiazhuang, 050031, Hebei, P.R. China

**Keywords:** Air enema, Intestinal mucosal immune function, Manipulative reduction, Pediatric intussusception

## Abstract

**Objective:**

To explore the effect of manipulative reduction combined with air enema on intestinal mucosal immune function in children with intussusception.

**Methods:**

This is a prospective randomized controlled study in which 60 children with primary intussusception admitted to Hebei Children’s Hospital from October 2018 to October 2019 were selected for this study. They were randomly divided into two groups. The 30 patients in the experimental group underwent manipulative reduction and air enema reduction, and 30 patients in the control group underwent only air enema reduction. Pain scores and pressure during enema were recorded and analyzed. Fasting blood of children in the experimental group were drawn to test the serum T lymphocyte subsets CD3+, CD4+, CD8+ levels, B lymphocyte subsets CD19+ level, and NK cell subsets CD56+ levels before reduction. Among them, fasting blood of 28 children with successful reduction were drawn again in the morning after reduction, and the indicators of each immune cell subgroup before and after reduction were analyzed. Two children with unsuccessful reduction were no longer tested for these indicators.

**Results:**

Twenty-Eight children in the experimental group had successful reduction, and two children with unsuccessful reduction were changed to open surgery (28/30). Twenty five Children in the control group had successful reduction, and five were changed to open surgery (25/30). There was no significant difference in the success rate of reduction between two groups (p>0.05). Close observation for 12~24h after reduction found that none of the children had signs of peritonitis. The pain score and reduction pressure of the observation group were lower than those of the control group, and the difference was statistically significant (p<0.05). The levels of serum CD3+, CD4+, and CD8+ after reduction in the experimental group were significantly higher than before reduction, and the difference was statistically significant (p<0.05). CD19+ level was significantly lower than before reduction, and the difference was statistically significant (p<0.05). There was no significant difference in changes of other indicators.

**Conclusions:**

Manipulative reduction combined with air enema reduction can relieve pain and air injection pressure during enema, reduce reperfusion injury caused by intestinal ischemia, and protect intestinal mucosal immune function, which is a favored treatment.

## INTRODUCTION

Intussusception is a common pediatric disease requiring emergency treatment. The incidence rate of intussusception is about 2.3%, and in severe cases, it is life-threatening to children.^1^ Intussusception refers to a certain section of intestine and the corresponding mesentery fold into the adjacent intestinal cavity, most of which occur in infants and young children, under two years old.^2^ According to the ways of intestinal tubes folding, intussusception can be classified into small intestine-small intestine type, small intestine-colon type, colon-colon type, and ileum-colon type intussusception is most common in children.^3^ Intussusception develops rapidly, may lead to serious complications such as intestinal ischemic necrosis, intestinal perforation, peritonitis, and even death.^4^ Air enema is currently recognized as a reliable and effective non-surgical treatment method for intussusception in children, but children experience great pain during the reduction process.^5^ In addition, high air pressure in the intestine may cause intestinal perforation, ischemia-reperfusion injury, and peritonitis.^6^ Our objective was to explore the effect of manipulative reduction combined with air enema on intestinal mucosal immune function in children with intussusception

## METHODS

.Sixty Children with primary intussusception admitted to Hebei Children’s Hospital from October 2018 to October 2019 were selected for this study.

### Inclusion criteria:

1. Children met the diagnostic criteria for primary intussusception and the indication for air enema.
2. Conscious children without facial paralysis or disability.
3. Informed consent of family members.

### Exclusion criteria:

1. Children with mental abnormality or consciousness disorder.
2. Children underwent unsuccessful reduction and needed operation.

### Ethical approval

The study was approved by the Institutional Ethics Committee of Hebei Children’s Hospital (dated 23^rd^ June, 2019), and written informed consent was obtained from all participants.

Patients were grouped according to the date they were admitted to the hospital. Patients whose data of admitting to the hospital with odd number were put in the experimental group, and patients whose data of admission to the hospital were even number were put in the control group. thirty patients in the experimental group were treated with manipulative reduction combined with air enema reduction, and 30 patients in the control group were treated with just air enema reduction. The general information of children in the two groups were not statistically significant and comparable (Table-I).

**Table-I:** Comparison between patients’ general information of two groups ±S (n=30).

Groups	Number of Cases	Gender		Age (years)

		Male	Female	
Experimental group	30	18	12	1.86±0.97
Control group	30	16	14	1.83±0.92

### The experimental group

Patients in the experimental group were treated with manipulative reduction combined with air enema reduction. Manipulative reduction: used abdominal wall and bimanual examination to wrap the intussusception head, and moderately pushed in the direction of the retraction of the intussusception head. After reduction of a short section of the intussusception head, used air enema to slowly restore the intussusception tube. In cases of deep intussusception head, touched the end of the intussusception head by deep gliding palpation, tried to wrap the end of the intussusception head with fingertips and formed a bimanual examination with the left hand forwarding at the back waist, forming a wrap around the intussusception head. Did not increase pressure of the sheath from the intestinal cavity to the outside during the pushing process, and the abdominal wall around the sheath had a protective effect on the sheath. Once the reduction was unsuccessful, the patient should be ready to be sent to the operating room for surgical treatment. The method of air enema reduction was the same as used in the control group.

### The control group

Patients in the control group were treated with air enema reduction: (1) Before enema, anal occlusion was prepared to stimulate the anus for defecation. The rectum should be empty to the greatest extent, and the dilation of intestines and subphrenic free air were observed by thoracic and abdominal fluoroscopy. (2) The patient was in lithotomy position, and the 22th urinary catheter with lubricant applied to one end was inserted it into patient’s anus. 20ml of gas was injected into the gasbag. After fixation, patient was changed to the supine position, and the other end of the catheter was connected to the air enema machine which was adjusted to pulse mode; (3) Under the X-ray fluoroscopy, the pressure was slowly increased by the enema machine, the pressure was controlled at 6~13kpa. The intussusception in patient’s intestinal cavity was closely observed, surgical treatment was immediately performed once the reduction was failed.

### Evaluation indicators

The FLACC Pain Assessment Scale^7^ was used to evaluate the pain of patients, and the scores were scored by observing children’s facial and leg mobility, crying and consolable levels. Each item awarded 0~2 points, the total score was the sum of the scores of each item. The higher the score indicated greater the pain. Intussusception in the intestinal cavity was observed by X-ray, and the perfusion pressure during intussusception induction was recorded.

Evaluation of serum immune cell subset indicators before and after reduction in the experimental group. Fasting blood of children in the experimental group were drawn to test the serum T lymphocyte subsets CD3+, CD4+, CD8+ levels, B lymphocyte subsets CD19+ level, and NK cell subsets CD56+ levels before reduction. Among them, fasting blood of 28 children with successful reduction were drawn again in the morning after reduction, and the indicators of each immune cell subgroup before and after reduction were analyzed. Two children with unsuccessful reduction were no longer tested for these indicators.

### Statistical analysis

All data was analyzed by SPSS 20.0 software, and were expressed as (±S). Two independent sample t-tests were used in the data analysis between the experimental group and the control group, and the rate was compared using the χ^2^ test. In the experimental group, paired t test was used to compare and analyze the data of lymphocyte subsets before and after reduction. P <0.05 was considered statistically significant.

## RESULTS

Comparative analysis of children’s pain during enema between two groups (Table-II) suggested that the average pain score during enema in the control group was 6.63 ± 1.72, which was significantly higher than that in the experimental group, and the difference between the two groups was statistically significant.

**Table-II:** Comparison of children’s pain during enema between two groups (±S) n=30

Groups	Number of Cases	FLACC Score
Control group	30	6.83±1.72
Experimental group	30	5.43±1.25
t		8.55
p		<0.05

Comparison of children’s reduction pressure and success rate of air enema between two groups of patients (Table-III) suggested that the reduction pressure of the experimental group was significantly lower than that of the control group, the difference was statistically significant (p=0.00), and there was no significant difference in the reduction success rate between the two groups (p=0.65).

**Table-III:** Comparison of children’s reduction pressure and success rate of air enema between two groups (±S) n=30

Groups	Number of cases	Reduction Pressure (kpa)	Success rate (%)
Control group	30	10.37±1.52	83.3%(25/30)
Experimental group	30	7.04±1.23	93.3(28/30)
t/χ^2^		9.33	1.46
p		0.00	0.65

Analysis of changes in serum lymphocyte subsets in the experimental group before and after reduction (Table-IV) showed that the ratio of CD3+, CD4+, and CD8+ after reduction was significantly higher than before reduction. The difference was significant (p<0.05), and the ratio of CD19+ was significantly lower than before reduction, and the difference was significant (p<0.05).

**Table-IV:** Comparative analysis of serum lymphocyte subsets in the experimental group before and after reduction (*x̄*±S) n=28

Indicators	Before reduction	After reduction	t value	P value
CD3+	57.17±11.23	65.78±6.84	-3.515	0.002
CD4+	35.03±7.60	40.50±7.60	-2.610	0.015
CD8+	18.00±4.72	19.63±4.72	-2.662	0.013
CD4/CD8	2.12±0.82	2.24±0.83	-0.914	0.369
CD3+CD4-CD8-	5.00±2.67	5.33±3.48	-0.849	0.403
CD3+CD4+CD8+	0.10±0.11	0.12±0.14	-0.827	0.415
CD19+	29.14±9.84	22.51±6.44	3.147	0.004
CD56+	7.97±4.84	6.67±4.31	1.209	0.237

## DISCUSSION

Intussusception is a common abdominal emergency in children.^8^ It often occurs in infants and children under 1-year-old. Because infants and young children lack the necessary cognitive and expression skills, medical staff can only understand the disease according to parents’ description and children’s behavior and physiological response. During intussusception, because intestinal loop is a closed loop intestinal obstruction, generally the intestinal wall will have different degrees of spasticity and edema and blood circulation disorders.^9^ In severe cases, it may cause a series of surgical emergencies such as intestinal necrosis, peritonitis, and septic shock. Therefore, if considering the possibility of intussusception in children, emergency treatment is needed.^10^

The diagnosis and treatment of acute intussusception is mainly air enema, which is not only the diagnostic method of acute intussusception, but also the first choice of treatment for acute intussusception.^11^-^13^ It was reported that the success rate of air enema treatment of acute intussusception can be 90%.^14^ The pressure of the air enema is generally maintained at 6~13kpa. Starting from 6kpa, pulse inflation, exhaust and inflation are performed, and the pressure is gradually increased until the reduction is completed. Complications after reduction are mainly related to reduction pressure.^15^ The higher the reduction pressure, the higher the incidence of intestinal perforation, which is the most serious complication after reduction.^16^ It was found that the pressure during reduction using manipulative reduction combined with air enema reduction was lower than that of air enema alone, and the difference was significant (p<0.05). The reason for this could be that manipulative reduction uses the abdominal wall and bimanual examination to wrap the intussusception head and push it in the direction of the retraction of intussusception. The pushing process does not increase the pressure of the sheath from the intestinal cavity to the outside. Abdominal wall surrounding the sheath protects it and can also reduce the risk of bowel perforation in children. Most of children’s language ability is not yet developed, and the abdominal massage has a comporting effect when performing manual reduction, appropriately alleviating children’s violent confrontation and relaxing the abdominal muscles.^17^ It also reduced the intra-abdominal pressure, and relatively reduced the repulsive force caused by the increase of intra-abdominal pressure, so that the intussusception is more easily to reduction. The success rate of experimental was 93.3% (28/30), and the success rate of control group was 83.3% (25/30). Although there was no significant difference in this study, but it might due to the small sample size. For older children, medical staff can transfer their attention to relieve pain by language communication and physical contact during the process of reduction.

The intestinal mucosal barrier mainly consists of intestinal mechanical barrier and immune barrier.^18^ The intestinal mucosal immune barrier is mainly composed of secretory IgA (SIgA) and T lymphocytes in the lamina propria. Among them, two functional subgroups of T lymphocytes CD4 and CD8 respectively promote and inhibit local intestinal immunity. When CD4 and CD8 decrease, it leads to dysregulation of cell regulation, T cell-mediated cellular immunity is then suppressed, which ultimately leads to the body Intestinal immune function declines.^19^ Research have found that secretion of slgA and proliferation activity of related lymphocytes in the intestine were reduced in reperfusion injury due to intestinal ischemia, which was significantly negatively correlated with the extraintestinal tissue and plasma bacterial endotoxin translocation.^20^ It was speculated that injury of reperfusion after intestine ischemia, intestinal mucosal immune dysfunction may be one of the reasons for the translocation of bacterial endotoxin in extraintestinal tissue. The current intussusception reduction method, whether it is air enema reduction or liquid enema reduction, the common principle is to restore the intussusception by increasing the perfusion pressure. During the reduction process, the intestinal wall is squeezed to a certain extent, therefore, ischemic injury is inevitable, which in turn affects the immune function of the intestinal mucosa and leads to immune function disorder. This study confirmed that before intussusception reduction, serum CD4+, CD3+, CD8+, etc. were reduced to varying degrees. However, they were significantly improved on the second day after reduction compared with before reduction. This indicated that manipulative reduction combined with air enema reset had lower pressure and less painful stimulation, which can effectively reduce reperfusion injury due to intestinal ischemia and promote the recovery of intestinal immune function. In this study, manipulative reduction and air enema were combined to treat intussusception in children. Results indicated significantly reduced pain score, low perfusion pressure, slight injuries in intestinal mucosal ischemia due to reperfusion, and obvious restore of the mucosal immune protection mechanism.

### Limitations of the study

(1) According to different age, different course of disease, stratified research may be more conducive to clinical guidance; (2) the study had a small sample size, short follow-up period and no more detailed subgroup comparison. Our findings were still needed to be further confirmed by more in-depth studies in the future.

## CONCLUSION

Manipulative reduction combined with air enema can relieve pain, reduce pressure and reperfusion injury due to intestinal ischemia, and have a fast recovery of intestinal mucosal immune function, which is a good treatment for children with intussusception.

### Authors’ Contributions:

**YL and** **PW** designed this study and prepared this manuscript, and are responsible and accountable for the accuracy or integrity of the work.

**HLJ** collected and analyzed clinical data.

**YKB** significantly revised this manuscript.
